# Effects of Rare Earth Metal Promotion over Zeolite-Supported Fe–Cu-Based Catalysts on the Light Olefin Production Performance in Fischer–Tropsch Synthesis

**DOI:** 10.1021/acsomega.2c05795

**Published:** 2022-12-27

**Authors:** Utku Burgun, Hadi R. Zonouz, Hasancan Okutan, Hüsnü Atakül, Selim Senkan, Alper Sarioglan, Gamze Gumuslu Gur

**Affiliations:** † Chemical Engineering Department, Istanbul Technical University, 34469Istanbul, Turkey; ‡ ITU Synthetic Fuels and Chemicals Technology Center, ITU-SENTEK, 34469Istanbul, Turkey; § Chemical and Biomolecular Engineering Department, University of California, Los Angeles, Los Angeles, California90095, United States

## Abstract

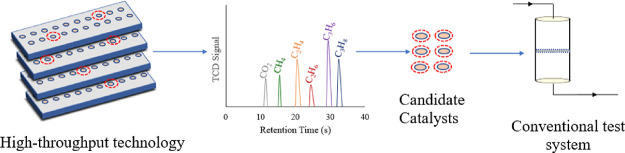

Fischer–Tropsch synthesis (FTS), a significant reaction for effective H_2_ utilization, is a promising approach for direct production of light olefins from syngas (H_2_ + CO). For the FT-Olefin process, an efficient catalyst restricting the product distribution of FTS to light olefins is required. Aligned with this goal, we synthesized 24 catalysts comprising Fe and Cu in combination with rare earth metals (La, Ce, Nd, Ho, Er) and zeolite supports (ultrastable Y and mordenite). FT-Olefin performances of these catalysts were screened using a high-throughput test system at atmospheric pressure, and then promising catalysts were tested under high pressure in a conventional test system. Results show that Nd increases selectivity to light olefins and Ho suppresses C_5_+ and coke formation. It is also demonstrated that zeolite–metal interaction, leading to a mixture of both acidic and basic sites, is significant in increasing light olefin production. The mordenite-supported 20 wt % Fe, 0.5 wt % Cu, and 0.5 wt % Ho catalyst provides the highest light olefin yield with the lowest coke and heavier hydrocarbon selectivity.

## 1 Introduction

Light olefins (C_2_^=^-C_4_^=^) are significant key building units mostly used in the petrochemical industry and production of various chemicals that are widely used in daily life and in industrial applications.^1^ The conventional method of light olefin production is via steam cracking of the petroleum product naphtha.^2^ However, depletion of petroleum reserves necessitates finding alternative routes that facilitate other sources such as coal or biomass for production of value-added products. One of the promising alternative routes is the Fischer–Tropsch to olefin (FT-Olefin) synthesis in which syngas (CO + H_2_) obtained from gasification of coal, biomass, etc., can be directly converted into light olefins without any additional steps.^1,3,4^ Fisher–Tropsch synthesis (FTS) is a polymerization-like reaction in which elementary building units, CH_x_, combine to produce a wide range of hydrocarbons (C_1_-C_40_) through the chain growth mechanism.^2,5^ Both olefin- and paraffin-type products are formed as a result of hydrogenation and dehydrogenation reactions. In addition to these reactions, the presence of alcohol production, water gas shift (WGS), and Boudouard reactions complicate the nature of FTS. Therefore, FT-Olefin synthesis requires a strict control of reactions in FT synthesis to restrict the product range to olefins. This could be achieved by developing proper and efficient FT-Olefin catalysts.^2,6^ This stands as the main challenge to the commercial application of FT-Olefin in the industry.

In catalyst synthesis, Ru, Rh, Ni, Fe, and Co are generally chosen as the active metals because of their high activity for FTS. Among these metals, Fe is commonly used as the active metal in FT-Olefin synthesis because of its higher selectivity to light olefins, lower selectivity to methane, and lower cost.^1,3,4,7,8^ Another important criterion in developing FT-Olefin catalysts is the choice of the support material. To prevent active metal agglomeration that leads to activity loss and to increase active metal dispersion, various materials including metal oxides such as TiO_2_, Al_2_O_3_, ZrO_2_, carbon-based materials, and zeolites are used as support.^2,3^ Zeolites are considered as noteworthy support materials because of their adjustable Si/Al ratio, pore size, and channel dimensionality. The Si/Al ratio defines the acidity of zeolites.^9,10^ Because of their acidic natures, zeolites not only are considered as support materials but also add functionality to the catalyst. This functionality leads zeolites to act like cocatalysts alongside the active metal.^11−14^ In the FTS literature, zeolites are mainly studied to obtain gasoline range products and waxes as they allow for the secondary hydrogenation of readsorbed lighter hydrocarbons. Thus, the literature on the use of zeolites for production of light olefins is scarce.^8,12,15−18^

The light olefin selectivity, activity, and stability of catalysts can be modified by adding small amounts of alkali, transition, and/or rare earth metals that act as promoters.^19,20^ Promoters can affect the catalytic performance in different ways; they may enhance the reduction of active metal by increasing H_2_ uptake or improve the dissociation of the CO molecule over the catalyst.^1,3,21^ Cu is one of the widely studied transition metals to observe the promoter effect in catalysts. It was reported that the presence of Cu sites on catalysts plays a significant role in the promotion of reduction of iron oxide enabling H_2_ spillover.^22,23^ Also, Cu is known to improve the surface basicity of the catalyst by its synergistic effect with alkaline metals leading to enhanced production of heavy hydrocarbons and olefins.^7,24^ Thus, the amount of Cu loading is critical with respect to the CO conversion and light olefin selectivity of catalysts. It was reported that increasing Cu loading from 2 to 5 (at.) % on a 100Fe/5.1Si/1.25K catalyst decreased the iron carbide formation rate, resulting in lower CO conversion and higher C_5_+ selectivity.^7^ In contrast to transition metals, rare earth metals (RE) were rarely studied as promoters on iron catalysts for FT-Olefin. The electronegativities of rare earth metals are lower than that of Fe; thus, they could donate electrons to iron atoms, leading to an enhanced surface electron density of iron atoms.^25^ The elevated electron density of the iron surface not only facilitates the CO adsorption and the formation of the Fe–C bond, which is required for activity, but also restrains the hydrogenation reaction by hindering the formation of the Fe–H bond.^25^

In this study, we used ultrastable Y (USY, Si/Al = 80, H^+^ form) and mordenite (MOR, Si/Al = 20, NH_4_^+^ form) zeolites with their well-defined pore structures as support materials combined with rare earth metal promoters, La, Ce, Nd, Ho, and Er, to prepare 24 different Fe–Cu based catalysts. One of the aims of the study was to observe if the zeolite-active metal-promoter functionality could suppress the secondary hydrogenation activity, shifting FTS product distribution toward light olefins. Therefore, two zeolites (USY and MOR) with different structures, porous networks, and Si/Al ratios were selected. To show the functionality of the zeolite as a support material, an additional catalyst was prepared using activated carbon (AC) as support because of its high specific surface area, its inert surface, and especially its weak interaction with the active metals.^26,27^ Rapid olefin production performance screening of the zeolite-supported 24 catalysts was conducted using a high-throughput catalyst performance analysis (HT-CPA) test system that operates at atmospheric pressure. High-throughput techniques have been successfully used for the development and discovery of new catalysts in the last decade, as they allow for high-speed performance screening of catalysts.^28,29^ However, the high-throughput catalyst screening technique has not been explored for FT-Olefin synthesis yet. It was our aim to show for the first time in the literature that the HT-CPA system can be successfully used to determine promising FT-Olefin catalyst candidates. For this purpose, catalyst candidates determined in the atmospheric pressure HT-CPA system were also tested under realistic FT conditions at high pressure and characterized by X-ray diffraction (XRD), H_2_-temperature programmed reduction (H_2_-TPR), NH_3_-temperature programmed desorption (NH_3_-TPD), CO_2_-temperature programmed desorption (CO_2_-TPD), Fourier Transform infrared spectroscopy (FT-IR), N_2_ adsorption, and elemental analyses. It was concluded that the HT-CPA and pressurized test system analysis results of selected catalysts were well correlated. The Ho-promoted MOR-supported 20Fe0.5Cu0.5Ho/MOR catalyst showed the highest olefin yield (0.47 × 10^–4^ g C/g Fe·s) with 21% olefin selectivity. The same catalyst formula supported on AC proved the functionality added by MOR zeolite to the catalyst in enhancing olefin production. The olefin yield and selectivity dropped to almost 0, and the product distribution shifted toward methane and C_2_-C_4_ paraffins on the AC support. Experimental results and the literature lead us to conclude that Fe–Cu–RE impregnated zeolites act like a bifunctional catalyst where the paraffins are formed on the active metal sites and dehydrogenated to olefins on the acid sites of the zeolites.

## 2 Results and Discussion

### 2.1 Screening of Catalysts

Conventionally, very long test durations (usually for days) are required to investigate the performance of catalysts in FT synthesis. Fortunately, high-throughput testing allows the accelerated discovery of FTS catalysts. The HT-CPA system used in this study is designed for fast catalyst screening, reducing the time and resource expenditure, but it operates under atmospheric pressure, far from the conventional FTS conditions. To be more precise, HT-CPA performs at atmospheric pressure with much higher gas hourly space velocities in the range of 20 NL/h·g compared to the reference values of 10–30 bar pressures and 1–3 NL/h·g for conventional FTS. Therefore, this necessitates elaborate interpretation of the HT-CPA data. On the other hand, it is an advantage that the given operating conditions render CO conversions well below 15% and thereby do not allow extensive coke formations.

In an effort to understand the effect of rare earth metals on hydrogenation, an activity performance indicator in HT-CPA data is needed. In search of a reasonable performance indicator, hydrogenation reactions over FT catalyst were evaluated on the basis of the proposed carbide mechanism.^30^ In this mechanism, C–O bond cleavage leads to the formation of C_1_ species on the surface. If the hydrogenation rate on the metal surface is high, this C_1_ species is fully hydrogenated and leaves the surface as CH_4_. If the hydrogenation rate is low, the C_1_ species is partially hydrogenated to the CH_x_ species that is responsible for the chain growth. Hence, for FT-Olefin synthesis, a relatively lower hydrogenation rate is required. In this sense, it was deduced to use the molar ratio of C_2_-C_3_ olefins to CH_4_ to correlate with catalytic hydrogenation.

Performance screening of 24 catalysts was carried out in HT-CPA using the methods previously described. Because both the process conditions and the catalyst formulation are effective in determining the olefin to paraffin ratio (O/P) for a given chain length, each of the microchannel reactors was operated under the same conditions for the accuracy of comparison of the catalytic activities. HT-CPA screening results are shown in Table 1 on the basis of the molar ratio of C_2_-C_3_ olefins to CH_4_. The results given in the table show that, at first glance, the addition of Cu to USY did not lead to a remarkable change on the (C_2_^=^-C_3_^=^)/CH_4_ ratio, unlike the MOR-supported catalyst on which higher (C_2_^=^-C_3_^=^)/CH_4_ ratios were obtained for lower Cu loads. To find an explanation, similar studies were examined for zeolite-supported Cu. In a study by Lappas et al.,^31^ a marked increase in gaseous alkene formation was achieved by partially Cu exchanged HZSM-5, and this was ascribed to the optimum balance between the dehydrogenation activity of the metal and acid function of the shape selective HZSM-5. Because the MOR used in this study is richer in aluminum compared to the USY, the interaction between its acid centers and the active metal of the catalyst would be more precise and would have a greater effect on olefin production.

**Table 1:**
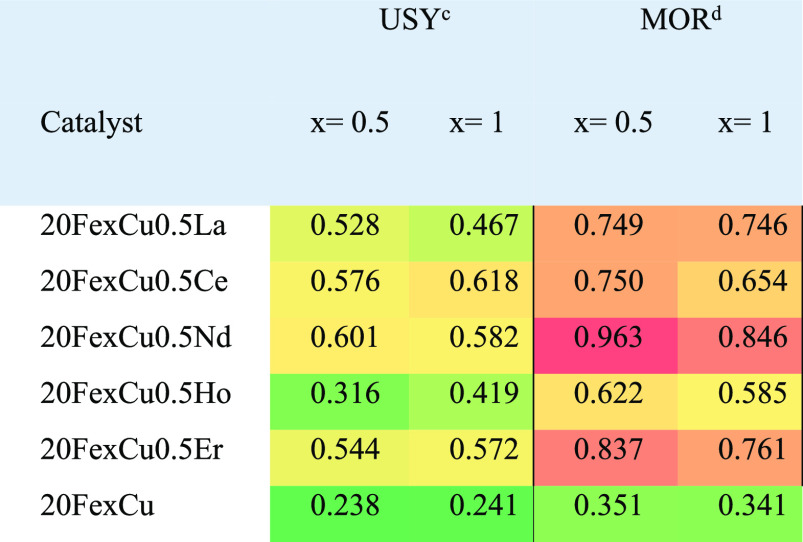
FT-Olefin Performance of Catalysts in HT-CPA as Indicated by the C_2_^=^-C_3_^=^/CH_4_ Ratio^a^^,^^b^

a Reduction conditions: *T* = 350 °C, *P* = 1 bar, H_2_/N_2_ = 1:1. Reaction conditions: *T* = 310 °C, *P* = 1 bar, H_2_/CO = 2:1.

b Color coding: green, yellow, and red indicate low, medium, and high values, respectively.

c USY, Si/Al = 80, H^+^ form.

d MOR, Si/Al = 20, NH_4_^+^ form.

Besides Cu, the effects of rare earth elements and the type of zeolite were clear. As seen in Table 1, the MOR-supported catalyst promotes olefin selectivity more, regardless of the catalyst composition. It is well known that the shape selectivity and the acidity, which are the two characteristic features of zeolites, give the bifunctionality to the final catalyst and thus manipulate FT product distribution remarkably. The shape selectivity of a zeolite comes from its pore structure, which is unique to each kind and adds on for the encapsulation of metal clusters. On the other hand, zeolite acidity is a result of its aluminum content and dictates its hydroisomerization and hydrocracking function. To understand the role of the zeolite on the overall catalytic behavior of cobalt-based Fischer–Tropsch catalysts, zeolites have been well discussed by Bessell,^32^ Sineva et al.,^12^ and Fraenkel and Gates.^33^ In line with the discussions of the aforementioned authors, it is clear that active metal sites catalyze FTS to produce hydrocarbon products and that their further reactions take place over transformed zeolite acid sites. It can be stated that shorter-chain hydrocarbons are favored in excess of acid sites. As mentioned in a study by Rahimi and Karimzadeh,^34^ to explain the acid catalyzed cracking of hydrocarbons, it was proposed that medium pore size and high-silica zeolites having acid sites few in number but strong enough might promote alkanes (mostly methane and ethane). It was also added that the yield of methane and chain growth probability decrease as the distance between the acid sites and metal sites gets closer, i.e., for high acid site concentrations.^12,34^ In line with this, it might be reasonable to obtain a high (C_2_^=^-C_3_^=^)/CH_4_ ratio over MOR possessing more acid centers compared to USY.

In addition to the deterministic role of zeolite structure and its physicochemical properties on FT catalyst activity, a positive effect of rare earth promotion was observed for all catalysts with respect to their olefin selectivity. The highest increase in (C_2_^=^-C_3_^=^)/CH_4_ ratio was obtained for the Nd-promoted catalyst, whereas the least was recorded for Ho. Wakui et al.^35^ investigated the cracking of *n*-butane over rare-earth HZSM-5 catalysts and reported that the formation of aromatic and heavier products was suppressed thanks to the inhibited readsorption of olefins. As reported by Xiaoning et al.,^36^ light rare earth metals, namely, La, Ce, Pr, Nd, Sm, Eu, and Gd, as promoter for HZSM-5 catalyst greatly enhanced its selectivity toward light olefins for the catalytic cracking of butane. The best total alkene yield was achieved over Ce- and Nd-promoted catalysts. They explained this improvement for rare earth doped zeolite catalysts as coming from their altered acidic properties and the existence of empty f orbitals of rare earths favoring the formation of Lewis acid sites.^36^ All these were evaluated to be consistent with the results given in Table 1. Therefore, in explaining the performance of catalysts used in this study, the behavior of catalysts (both the promoters and the supports) in dehydrogenative cracking of *n*-butane can be considered to be analogous to FT reaction tuned for light olefin production.

Up to this point, we showed that HT-CPA can be used for rapid FT catalyst discovery. Taking a further step, six catalysts were chosen for catalytic performance studies under more realistic FT conditions, namely, at high pressures with reasonable gas hourly space velocities. The chosen catalysts were 20Fe0.5Cu/USY and 20Fe0.5Cu/MOR as the reference case, 20Fe0.5Cu0.5Ho/USY and 20Fe0.5Cu0.5Ho/MOR as Ho leads to the lowest olefin production, and 20Fe0.5Cu0.5Nd/USY and 20Fe0.5Cu0.5Nd/MOR as Nd leads to the highest olefin production among the rare earth promoted ones in HT-CPA analyses.

### 2.2 Characterization Analyses

Catalysts selected at the end of HT-CPA screening tests were characterized using a series of different techniques to understand the possible source of differences observed in FT-Olefin performances of catalysts.

Calculated textural properties of supports and catalysts based on N_2_ adsorption analysis data are given in Table 2. Differences in the 3D structures and pore structures of USY and MOR zeolites act as the main reason for the lower (higher) surface area and pore volume of the MOR (USY)-supported catalysts. This could also be the reason why the addition of Cu, Ho, or Nd did not affect the final textural properties of the catalysts on USY support as opposed to MOR. The surface area of the 20Fe0.5Cu/MOR catalyst decreased significantly with Ho addition, whereas it increased with Nd addition. Similarly, addition of 20Fe0.5Cu and 20Fe0.5Cu0.5Ho to MOR caused a decrease in pore volume, whereas Nd had a diverse effect and caused pore volume to increase to a value even higher than that of MOR.

**Table 2:** Textural Properties of Supports and Fresh (Calcined) FeCuRE/Zeolite Catalysts

Catalyst	BET surface area (m^2^/g)	Pore Volume (cm^3^/g)	Micropore Volume^a^(cm^3^/g)	Mesopore Volume (cm^3^/g)
USY	716	0.513	0.228	0.285
20Fe0.5Cu/USY	481	0.376	0.145	0.231
20Fe0.5Cu0.5Ho/USY	464	0.361	0.146	0.215
20Fe0.5Cu0.5Nd/USY	472	0.372	0.155	0.217
MOR	301	0.194	0.146	0.048
20Fe0.5Cu/MOR	226	0.184	0.102	0.082
20Fe0.5Cu0.5Ho/MOR	136	0.167	0.061	0.106
20Fe0.5Cu0.5Nd/MOR	274	0.233	0.127	0.106
AC	698	0.747	0.177	0.570
20Fe0.5Cu0.5Ho/AC	497	0.462	0.130	0.332

a Calculated from the t-plot.

It is common knowledge that Si/Al ratios of zeolites govern both their thermal and chemical stability; i.e., the higher the ratio is, the higher is the stability. In a study conducted by Henriques et al.,^37^ three samples of Y zeolites with different rare earth contents were studied, and it was shown that whereas the global silica to alumina ratio (SAR) of all samples was around 5.3, the SAR of the framework is much lower for the high rare earth comprising zeolite, an indication of the improved stability. This was attributed to the less extended dealumination of the framework under hydrothermal conditions owing to the formation of RE–O–RE bonds. These bonds are responsible for the stabilization of the framework forming bridges with structural tetrahedra. Because trivalent rare earth cations neutralize three acid sites, aluminum-rich MOR is more susceptible to rare earth stabilization than USY. This interaction of RE with MOR was also observed through FT-IR analysis, given in Figure S1. While no significant differences were observed between the FT-IR spectra of fresh and spent USY catalysts, it was observed that on MOR support, peaks in CO stretching and CH bending band region became more distinct in Nd- and Ho-promoted spent catalysts.

**Figure 1:**
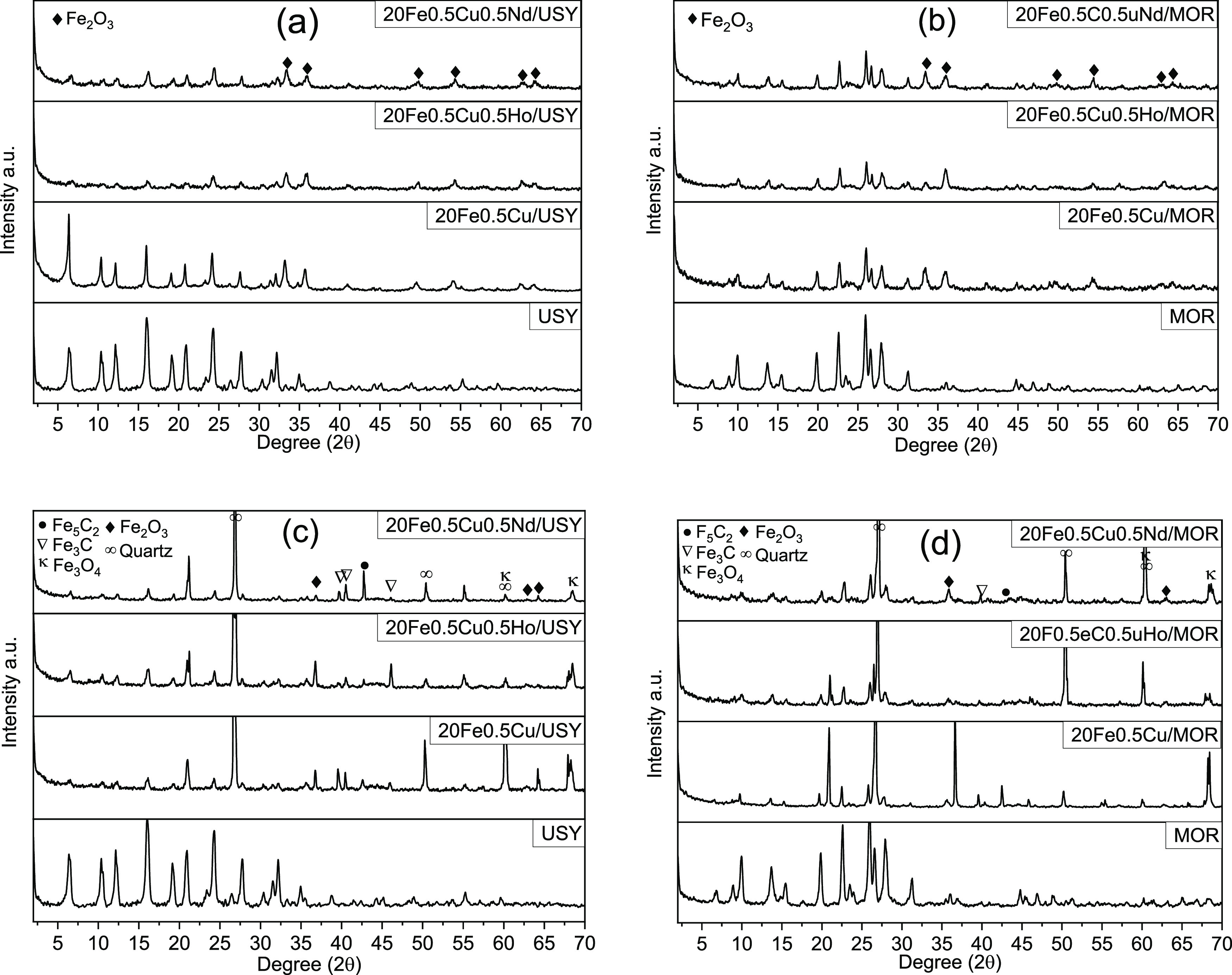
XRD profiles of the supports and 20Fe0.5Cu and 20Fe0.5Cu0.5RE catalysts: (a) fresh catalysts on USY, (b) fresh catalysts on MOR, (c) spent catalysts on USY, and (d) spent catalysts on MOR.

XRD patterns of fresh (calcined) and spent zeolite-supported catalysts and 20Fe0.5Cu0.5Ho/AC are given in Figure 1a–d and Figure S2, respectively. The phase analysis of catalysts was done using the Phase Analysis Using Powder Diffraction software Match!. In Figure 1a,b, the diffraction peaks located at 2θ values of 33.32, 35.80, 49.71, 54.35, 62.76, and 64.15° (Match! entry: 96-901-5066) are used to determine the Fe_2_O_3_ phase for calcined catalysts.

The XRD patterns of spent catalysts show that after the reaction, iron carbide phases, Fe_3_C and Fe_5_C_2_, formed. The Fe_3_C phase was detected at 2θ values of 39.79, 40.62, and 45.97° (Match! entry: 96-100-8726), and Fe_5_C_2_ peaks were observed at the 2θ value of 42.75° (Match! entry: 96-152-1832). Carbide phase formation was more distinct on USY-supported catalysts compared to MOR-supported catalysts. The iron oxide phase, which was either reoxidized from the iron carbide phase or not carburized during the reaction,^38^ was also detected on spent catalysts. The absence of CuO_*x*_, HoO_*x*_, and NdO_*x*_ peaks suggests that these metals are either highly dispersed or cannot be detected because of their low amount.^39,40^

**Figure 2:**
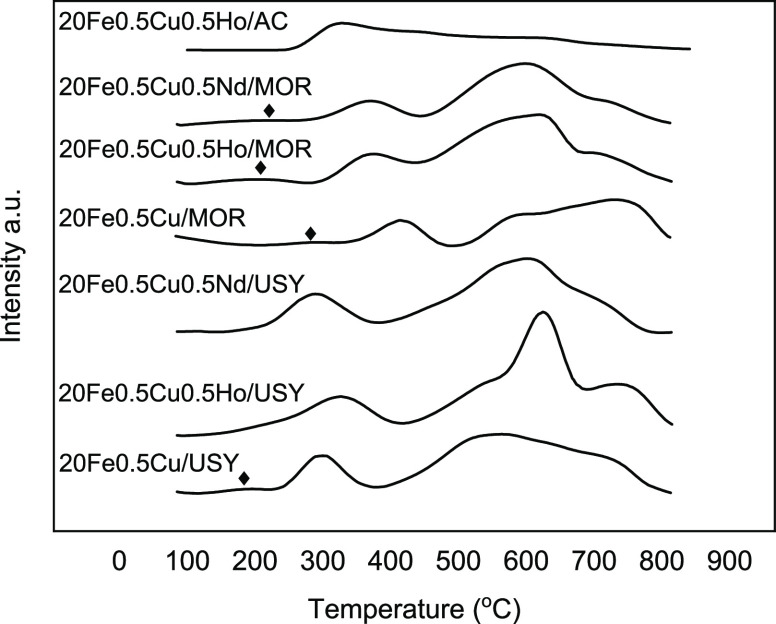
H_2_-TPR profiles of USY-, MOR-, and AC-supported catalysts. Black diamonds represent reduction of CuO to Cu. Individual TPR profiles of catalysts are given in Figure S3.

H_2_-TPR profiles of the zeolite- and AC-supported catalysts are given in Figure 2. The profiles display two peaks below 500 °C and one broad peak with a shoulder above 500 °C. The first peak is related to the reduction of CuO to Cu that generally occurs at temperatures below 250 °C.^41,42^ Reduction of Fe oxide occurs in multiple steps that correspond to peaks in the TPR profile. The peaks are attributed to the reduction of Fe oxide phases with respect to increasing temperature in the following order: Fe_2_O_3_ → Fe_3_O_4_ → FeO → Fe. Reduction of Fe_2_O_3_ (hematite) to Fe_3_O_4_ (magnetite) generally takes place at 320–380 °C, whereas reduction of Fe_3_O_4_ to FeO (wüstite) and metallic Fe generally occurs above 500 °C.^43,44^ On the other hand, from a thermodynamic point of view, iron oxide reduction can proceed at low temperatures. If the reduction temperature is lower than 570 °C, reduction occurs in two steps (Fe_2_O_3_ to Fe_3_O_4_ and Fe_3_O_4_ to α-Fe).^45,46^ In terms of kinetics, increasing the reduction temperature will facilitate the reduction of iron oxides as well. That is why, at a reduction temperature of 350 °C, both magnetite (Fe_3_O_4_) and α-Fe phases are reasonably expected.

CuO to Cu reduction can be observed by a separate peak as marked in TPR profiles of MOR supported catalysts and 20Fe0.5Cu/USY catalyst (Figure 2). The overlapping of Fe and Cu reduction peaks is attributed to well dispersion of the Cu phase,^13,47^ which suggests that Cu was well dispersed on Ho- and Nd-promoted USY-supported catalysts. In addition, Ho and Nd promotion on USY support led to shifting of the peak temperature in opposite directions. However, on MOR support, both Ho and Nd promotion decreased the first reduction temperature. The first TPR peak temperature of FeCuHo/USY was 325 °C and shifted to 369 °C for FeCuHo/MOR. The remaining TPR peak temperatures of these catalysts extending at the high-temperature region, namely, between 450 and 800 °C, were almost the same. However, these peaks were sharper for FeCuHo/USY compared to FeCuHo/MOR. The same behavior was observed among FeCuNd/USY and FeCuNd/MOR and FeCu/USY and FeCu/MOR. This and the fact that the reduction temperatures of MOR-supported catalysts are higher than those of USY-supported catalysts can be attributed to MOR–Fe interaction due to the higher aluminum and thus acidity content of MOR compared to USY.^13^ As reported in the literature, the use of alumina and silica supports may lead to formation of inactive iron species that then suppress the reducibility of iron oxide as a result of alumina and/or silica iron interaction.^43,48^

**Figure 3:**
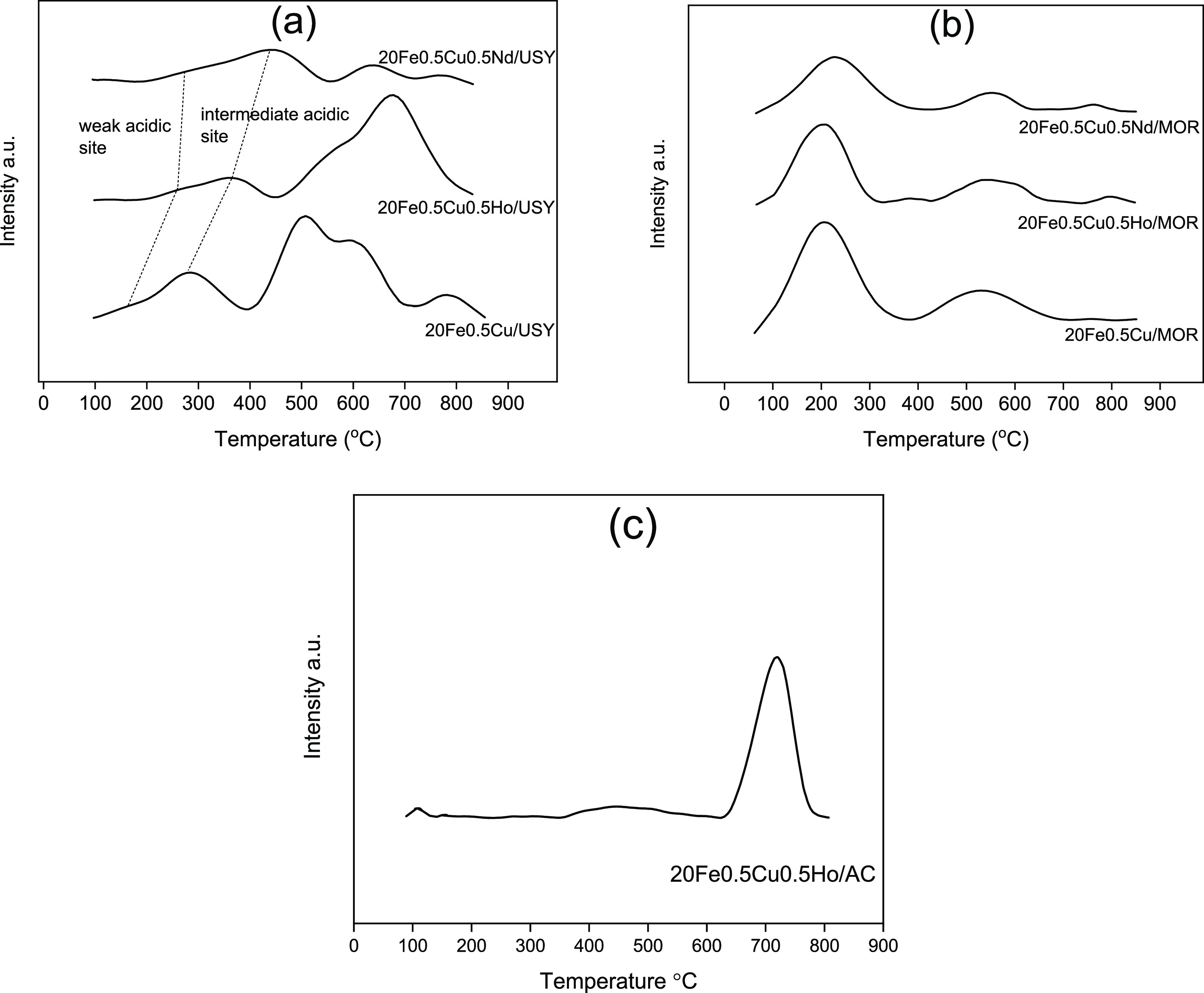
NH_3_-TPD profiles of (a) USY-supported catalysts, (b) MOR-supported catalysts, and (c) AC-supported catalyst.

The NH_3_-TPD profiles and related acid concentrations of catalysts are given in Figure 3a–c and Table 3, respectively. NH_3_-TPD profiles of USY and MOR supports are given in Figure S4a. The total amounts of acidic and basic sites of USY and MOR supports are given in Table S1. In USY-supported catalysts, the peaks in the temperature range of 100–350, 350–500, and above 500 °C are responsible for the weak, intermediate, and strong acidic sites, respectively.^49^ The shift observed in the first two peak temperatures to higher values indicates that Ho and Nd promoters increase the strength of weak and intermediate acidic sites on the 20Fe0.5Cu/USY catalyst.^50^ On the other hand, the overall acid concentration on USY-supported catalysts did not change significantly upon Ho promotion but decreased drastically by introduction of the Nd promoter. In contrast to USY-supported catalysts, weak acid site concentration of 20Fe0.5Cu/MOR catalysts decreased with the addition of Ho and Nd, which could be attributed to the higher aluminum content of MOR. Because Nd addition stabilizes the framework Al of USY and MOR zeolites as described above, it is reasonable that a lower amount of total acid sites occurs in Nd-promoted catalysts.

**Table 3:** Surface Acidity of USY- and MOR-Supported Catalysts Measured by NH_3_-TPD

	acidic site (μmol NH_3_/g cat)			
catalyst	weak acidic site	intermediate acidic site	strong acidic site	total acid sites
20Fe0.5Cu/USY	2.06	52.39	183.67	238.12
20Fe0.5Cu0.5Ho/USY	16.99	13.25	183.31	213.55
20Fe0.5Cu0.5Nd/USY	6.62	54.48	75.06	136.16
				
20Fe0.5Cu/MOR	1463.13		466.99	1930.12
20Fe0.5Cu0.5Ho/MOR	1043.19		549.48	1592.67
20Fe0.5Cu0.5Nd/MOR	766.42		188.95	955.37
				
20Fe0.5Cu0.5Ho/AC				412.4

The acidity obtained on different zeolites can also be related to their stabilities. As mentioned previously, zeolite stability increases with RE addition. It is claimed in majority of the studies that the RE ions move into the ion-exchange site, and from there, they exert a stabilizing effect by electrostatic interaction.^51^ This interaction is mostly related to the formation of RE–O–RE bonds inside the zeolite cavities through the formation of bridges with structural tetrahedra. As was indicated by Bolton,^52^ RE exchanged Y zeolite exhibits two-thirds of the acidity of dealuminated Y. As discussed in the manuscript, acid sites over zeolite are partly neutralized, and the framework was stabilized with RE addition. As an aluminum-rich zeolite, MOR was more prone to the neutralization effect of rare earths as a measure of decreased total acidity (Table 3). Meanwhile, USY, which as supplied was formerly in an already stabilized state, has quite lower total acidity values, and hence, the effect of RE stabilization is not visible. When the zeolite was not stabilized, immobile nonstructural aluminum produced by calcination remains inside the zeolite pores as stated by Miller et al.^53^ Therefore, a decrease in total surface area and micropore volume could be expected for the less stable forms of zeolite frameworks. When the values given in Tables 2 and 3 were examined, the stabilization effect was clearly observed for Nd on aluminum-rich zeolite, namely, MOR. Higher micropore volume and lower total acidity were recorded for the FeCuNd/MOR catalyst compared to FeCu/MOR and FeCuHo/MOR ones. As a plausible explanation, immobile nonframework aluminum species might favorably form inside the cavities of the nonstabilized zeolite structure and might freely interact with iron and/or copper oxide species to form iron and copper aluminates. The aluminates might lead to plugging of micropores and a decrease in the total surface area. In opposition to Nd, Ho addition led to a decrease in total surface area and micropore volume when compared with FeCu/MOR. On the other hand, mesoporosity was improved and total acidity was decreased with Ho addition as well, in the same way as with Nd.

An overview of NH_3_-TPD results revealed that Nd-included catalysts acquired higher weak acidic site strength in comparison to the Ho-containing catalyst. This result might be related to the electronegativity of these promoters.^54^ The electronegativities of elements loaded on the catalysts decrease in the order of Cu (1.90) > Fe (1.83) > Ho (1.24) > Nd (1.14).^55^ Hence, the electronegativities of the catalysts are expected to decrease in the order of 20Fe0.5Cu > 20Fe0.5Cu0.5Ho > 20Fe0.5Cu0.5Nd on both supports. The TPD results showed that this decrease in electronegativity is aligned with the increase in weak site acidic strength, 20Fe0.5Cu < 20Fe0.5Cu0.5Ho < 20Fe0.5Cu0.5Nd.

**Figure 4:**
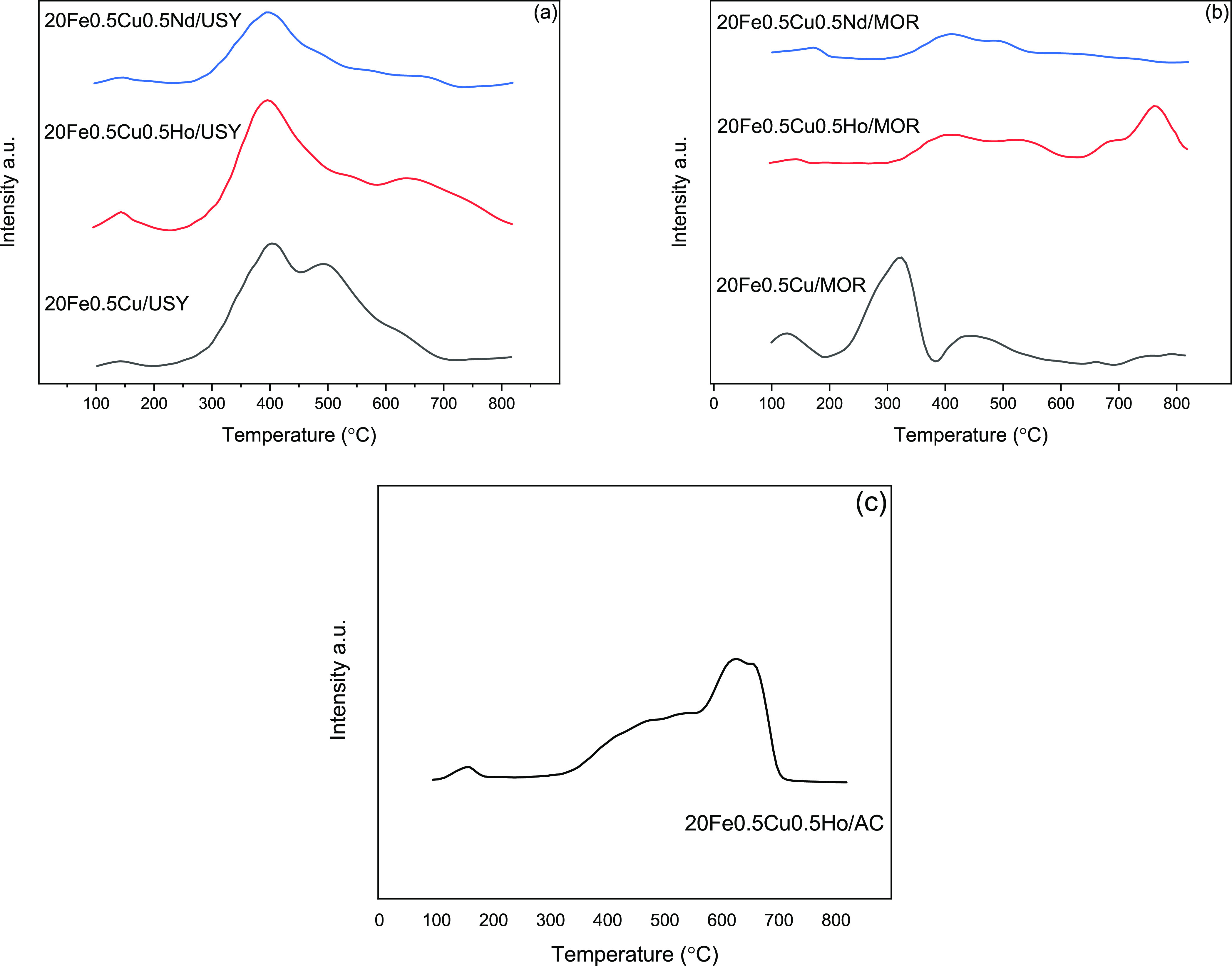
CO_2_-TPD profiles of (a) USY-supported catalysts, (b) MOR-supported catalysts, and (c) AC-supported catalyst.

The CO_2_-TPD experiments were performed to determine the basicity of calcined catalysts, and the profiles are given in Figure 4a–c. Figure S4b shows the CO_2_-TPD profiles of USY and MOR supports. In USY- and MOR-supported catalysts, desorption peaks were observed in the ranges of 100–200, 300–650, and above 650 °C that corresponded to weak, moderate, and strong basic sites, respectively.

As can be seen in Figure 4, the profiles, i.e., peak temperatures and intensities, change with the addition of RE metals. These changes are related to the differences in the strength and amount of basic sites on the catalysts, respectively. It can be seen in Table 4 that the total basicity of MOR-supported catalysts is lower than that of USY-supported catalysts. This result aligns with NH_3_-TPD experiments pointing out the higher acidity of MOR-supported catalysts. In addition, the total basic site amount on MOR-supported catalysts decreases upon promotion by RE metals, with Nd leading to the lowest concentration, on both supports. This is in agreement with the magnitude of the ionic radii of Nd (229 pm) and Ho (233 pm) as discussed in the literature;^56^ the total concentration of the basic sites decreases with the decrease in ionic radius of RE metals. In addition, just as observed on NH_3_-TPD experiments, Ho promotion causes an increase in the total site concentration on USY as opposed to its effect on MOR support.

**Table 4:** Surface Basicity of USY- and MOR-Supported Catalysts Measured by CO_2_-TPD

catalyst		weak basic site	moderate basic sites			strong basic sites		total (μmol CO_2_/g cat)
20Fe0.5Cu/USY	μmol CO_2_/g cat	2.62	163.81	63.57	54.04			284.04
	*T* (°C)	141	400	504	582			
20Fe0.5Cu0.5Ho/USY	μmol CO_2_/g cat	8.81	161.67	60.23		95.23		325.94
	*T* (°C)	144	393	500		650		
20Fe0.5Cu0.5Nd/USY	μmol CO_2_/g cat	1.2	78.81	49.04		8.09		137.14
	*T* (°C)	147	388	490		651		
								
20Fe0.5Cu/MOR	μmol CO_2_/g cat	5.48	60.23	34.05	31.19		9.52	130.95
	*T* (°C)	135	291	331	461		787	
20Fe0.5Cu0.5Ho/MOR	μmol CO_2_/g cat	2.14	32.14	34.76		13.57	46.42	82.61
	*T* (°C)	136	403	523		684	763	
20Fe0.5Cu0.5Nd/MOR	μmol CO_2_/g cat	3.33	29.52	10.95	14.28		0.95	58.01
	*T* (°C)	167	409	495	604		721	
								
20Fe0.5Cu0.5Ho/AC	μmol CO_2_/g cat	124.28	2242.38	1210.72				3577.38
	*T* (°C)	158	473	632				

### 2.3 Catalyst Performances in the Pressurized Test System

**Figure 5:**
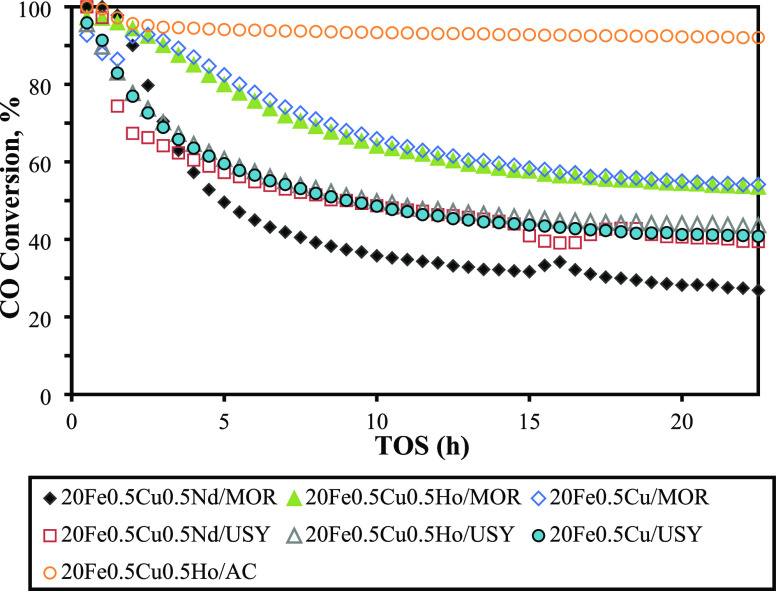
The activity (CO conversion, %) of USY-, MOR-, and AC-supported catalysts versus time on stream (TOS).

The catalysts selected based on HT-CPA screening results were tested for their performance under conventional FT conditions, i.e., at 310 °C and 10 bar with the inlet gas mixture of H_2_/CO = 2:1 for their FT-Olefin performance, using the methods described above. CO conversions of catalysts versus time-on-stream (TOS) data are shown in Figure 5, and all information regarding catalytic performance of catalysts is summarized in Table 5. A rapid initial loss of activity was observed in all catalysts except the AC-supported one. CO conversion trends of all USY-supported catalysts, whether promoted with rare earths or not, were very similar, whereas it was not the case for MOR-supported catalysts. Only the MOR-supported catalyst promoted with Nd was deactivated more severely as compared to unpromoted or Ho-promoted counterparts. At the end of 21 h, the highest conversions were achieved on 20Fe0.5Cu/MOR (55%) and 20Fe0.5Cu0.5Ho/MOR (54.5%) catalysts among all zeolite-supported catalysts. Coking was regarded as a possible explanation to the observed differences in deactivation. Therefore, carbon analysis of the spent catalysts were performed, and the amounts of carbon deposition on the zeolite-supported catalysts are reported in Table 6. These values point out that the degree of deposition is higher on MOR-supported catalysts and that RE promotion decreases carbon deposition. These results align with the acidity of the catalysts (Figures S5 and S6). The degree of carbon deposition is reported to be directly correlated with the acidity of the zeolite catalysts: an increase (decrease) in carbon deposition is observed with an increase (decrease) in acidity of the zeolite catalyst.^57,58^ However, it is only logical to compare the overall carbon amounts and acidities measured on USY- vs MOR-supported catalysts qualitatively rather than performing a one-by-one quantitative comparison. That is due to the fact that MOR and USY have completely different structures, and as partly discussed in HT-CPA test results, the zeolite structure and the strength of acid sites are both effective on coke formation.^59,60^ In this regard, the NH_3_ TPD analysis results of our catalysts (Table 3) revealed that MOR catalysts are more acidic compared to their USY-supported counterparts. Therefore, higher carbon deposition was expected on MOR catalysts. In addition, the acidic natures of both MOR- and USY-supported catalysts were more prominent before RE promotion. RE promotion has been reported to decrease the acidity and enhance coke resistance of the zeolites in other studies, in agreement with our results.^61,62^ Nevertheless, the carbon analysis results show that the deactivation of catalysts cannot be solely related to the carbon deposition on catalysts. This result suggests that other deactivation mechanisms such as sintering and/or iron reoxidation are more dominant in the observed activity loss. It is apparent that the USY-supported catalyst with large supercages linked tetrahedrally through pore openings of about 7 Å was more prone to deactivation than the MOR-supported catalyst that consists of main channels of 6.5 × 7.0 Å, which are connected by tortuous pores of 2.6 × 5.7 Å. Two-dimensional and somewhat narrower channels of MOR might provide a more confined space for active metals and restrict sintering.^63^ In addition, the XRD analysis of spent catalysts showed the presence of distinct Fe_2_O_3_ peaks on USY-supported catalysts and Nd-promoted MOR catalyst, which could indicate reoxidation of Fe sites leading to activity loss.

**Table 5:** Catalyst Performances^a^ in FTS at the End of 21 h in the P-CPA System

					hydrocarbon distribution (C %)						
catalyst	*X*_CO_ %	*S*_CO2_(% C)	*S*(C %) gaseous total HC	*S* (% C) coke/heavier HC	CH_4_	C_2_^=^-C_4_^=^	C_2_^o^-C_4_^o^	C_5_+	O/P	C_2_^=^-C_4_^=^ yield(g C/g Fe·s)(×10^–4^)	FT yield (mol CO converted/g Fe·s)(×10^–5^)
20Fe0.5Cu/USY	41.3	26.3	73.7	8.5	33.4	10.1	43.3	13.2	0.23	0.175	1.447
20Fe0.5Cu0.5Ho/USY	44.1	25.8	74.2	21.2	34.6	11.9	44.0	9.5	0.27	0.194	1.354
20Fe0.5Cu0.5Nd/USY	40.4	16.0	84.0	23.2	28.6	18.1	40.0	13.3	0.45	0.292	1.348
20Fe0.5Cu/MOR	55.0	33.4	66.6	11.1	32.1	20.4	31.7	15.8	0.65	0.418	1.702
20Fe0.5Cu0.5Ho/MOR	54.5	33.2	66.8	3.4	29.5	21.2	31.0	18.3	0.68	0.467	2.046
20Fe0.5Cu0.5Nd/MOR	28.3	23.3	76.7	14.8	34.9	25.4	27.1	12.6	0.94	0.290	0.952
20Fe0.5Cu0.5Ho/AC	92.2	37.0	63.01	17.2	47.3	0.4	44.7	7.6	0.01	0.013	2.502

a Reduction conditions: *T* = 350 °C, *P* = 1 bar, H_2_/N_2_ = 1:1. Reaction conditions: *T* = 310 °C, *P* = 10 bar, H_2_/CO = 2:1.

**Table 6:** Carbon Analysis Results of Spent Catalysts

catalyst	carbon wt %
20Fe0.5Cu/USY	2.6
20Fe0.5Cu0.5Ho/USY	1.6
20Fe0.5Cu0.5Nd/USY	1.1
20Fe0.5Cu/MOR	4.2
20Fe0.5Cu0.5Ho/MOR	2.8
20Fe0.5Cu0.5Nd/MOR	2.7

Light olefin selectivity with time on stream and at the end of 21 h of operation is presented in Figure S7 and Table 5, respectively. In line with HT-CPA screening results, C_2_^=^-C_4_^=^ selectivities over MOR-supported catalysts were higher than those on USY-supported ones. For example, C_2_^=^-C_4_^=^ olefin selectivity doubled from 20Fe0.5Cu/USY to 20Fe0.5Cu/MOR with or without Ho. In all cases, the highest olefin selectivity was achieved over the zeolite catalysts containing Nd. When each zeolite support was compared within themselves, the lowest CO_2_ and the highest gaseous hydrocarbon selectivity values were obtained in the presence of Nd; however, a similar effect was not observed with Ho.

To examine the effect of the rare earth element on FTS alone, ignoring the contribution of the zeolite support on the FTS reaction, AC was chosen for support. Although surface functional groups are present over AC, AC support interacts more weakly with metals dispersed over its surface than zeolites. Ho was deliberately chosen to see if any improvements were made because no major differences in selectivity were observed between the Ho and non-Ho zeolite samples. The AC-supported 20Fe0.5Cu0.5Ho catalyst was prepared and tested under the same conditions. As seen from Figure 5, ca. 92% CO conversion was achieved on 20Fe0.5Cu0.5Ho/AC. A high catalytic stability was observed as well. Product distribution of this catalyst given in Table 5 showed a high CO_2_ selectivity and almost equal production of CH_4_ and C_2_^°^-C_4_^°^ paraffins, almost 50% of each as seen in hydrocarbon distribution. However, almost no light olefin was obtained.

Selectivity to coke or heavier hydrocarbon formation, given in the fifth column of Table 5, was increased in the presence of rare earths except 20Fe0.5Cu0.5Ho/MOR. The increase in coke/heavier hydrocarbon selectivity was much more precise in USY catalysts. The carbon analysis results given in Table 6 represent the total carbon deposition on the surface of the catalysts throughout the FT reaction. Although it is not possible to quanitatively distinguish coke deposition and heavier hydrocarbon production amounts via this analysis, it enables qualitative comparison of heavy hydrocarbon production performances of catalysts. In that regard, on USY, it can be claimed that RE promotion led to an increase in heavier hydrocarbon selectivity, as the total carbon deposition decreased. The same effect was valid for Nd promotion on MOR-supported catalysts. However, Ho promotion led to a significant decrease in heavier hydrocarbon production on MOR support.

**Figure 6:**
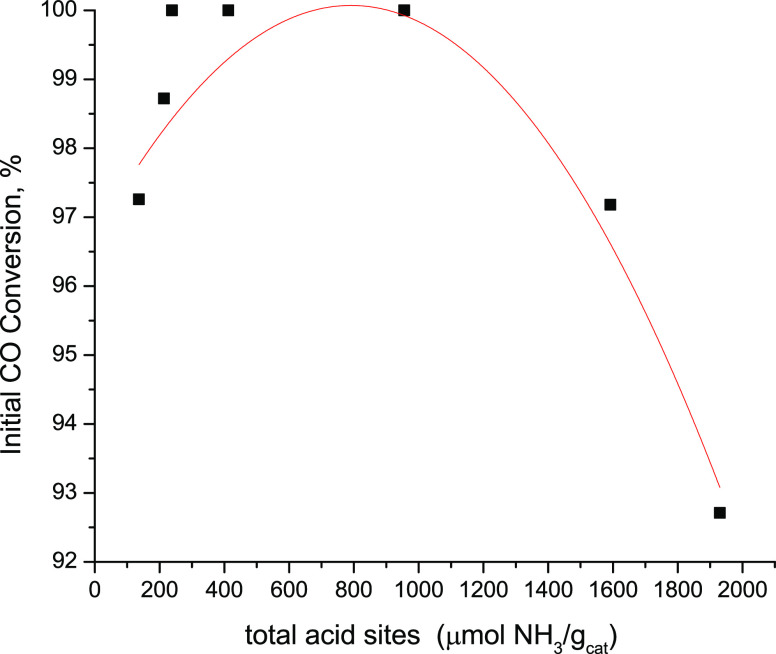
Initial CO conversion vs total acid sites.

**Figure 7:**
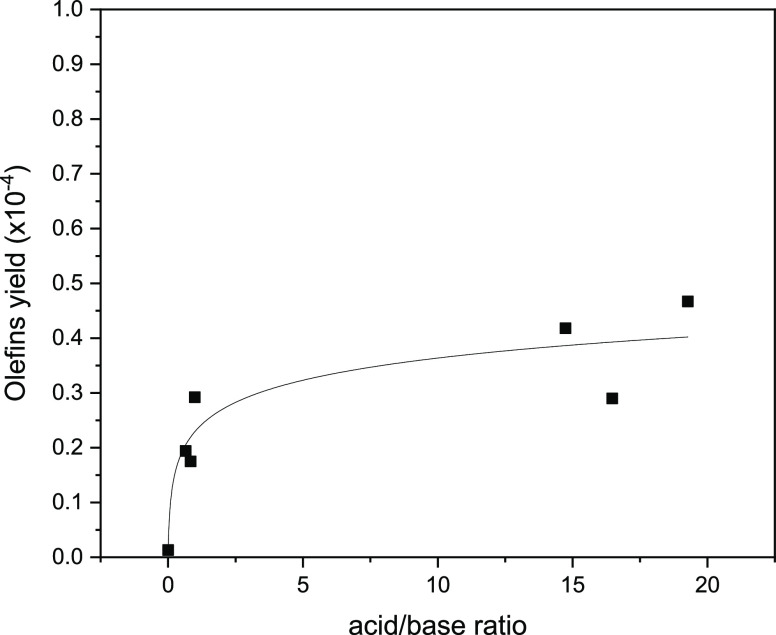
Olefin yield vs acid/base ratio of catalysts.

**Figure 8:**
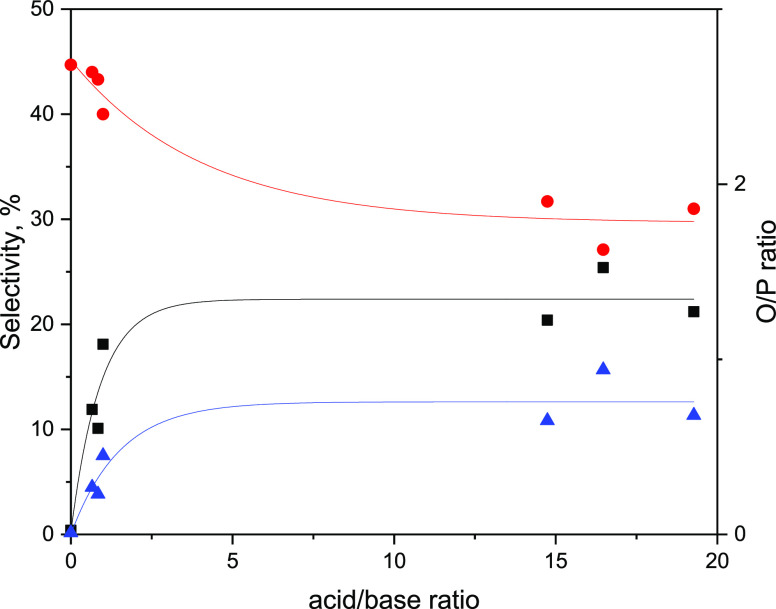
C_2_^=^-C_4_^=^ (black), C_2_^°^-C_4_^°^ (red) selectivity, and O/P ratio (blue) vs acid/base ratio.

In Table 7, the ratio of the amount of total acid sites to total basic sites is given. Figures 6–8 were obtained using acid concentration vs initial CO conversion and acidic/basic site ratios vs C_2_-C_4_ olefin yield, hydrocarbon selectivities, and O/P ratio. The initial CO conversion was observed to change with the acidity of the catalysts. As seen in Figure 6, initial CO conversion vs acidity has a volcano-like correlation. Light olefin yields were observed to correlate with the acid/base site ratio as shown in Figure 7. The light olefin yield shows a steep increase at low A/B values; however, this behavior was followed by a gradual increase as the A/B ratio increased more. Similarly, as seen in Figure 8, C_2_^=^-C_4_^=^ selectivity and O/P values increase with increasing A/B ratio. At low A/B ratios, O/P is more susceptible to changes in acid/base characteristics of the catalysts. However, above 2.5, the slope of the increase becomes gradual. This increase in C_2_^=^-C_4_^=^ selectivity accompanied with the decrease in C_2_-C_4_ paraffin selectivity leads to higher O/P with increasing A/B ratio. In the study by Prieto et al., the acid–base natures of Co-Ru-based catalysts prepared on Al_2_O_3_-based different metal oxide supports were estimated.^64^ They reported a dependence of the Fischer–Tropsch synthesis performance of the catalysts in terms of initial turnover frequency, C_4_, C_6_, and C_8_ olefin, and C_13_+ selectivities on the acid–base character of the catalysts. It was claimed that the acid–base nature of the support has a key role in controlling secondary reactions of olefins. The data obtained in this study, in alignment with the findings of Prieto et al., show the dependence of FTS performance on acid–base characteristics of the catalysts.^64^ The significance of acid–base natures of the catalysts was also shown in other studies: van Deelen et al.^65^ showed the relation between acid–base nature and yield, and Silvester et al.^66^ presented how the ratio of the number of acid and base sites correlates with the selectivity. Nevertheless, further experiments should also be performed to create more data points for the provided plots in this study.

**Table 7:** The Amount of Total Acid Sites^a^/the Amount of Total Basic Sites^b^ of Catalysts

catalysts	acid/base ratio
20Fe0.5Cu/USY	0.838
20Fe0.5Cu0.5Ho/USY	0.655
20Fe0.5Cu0.5Nd/USY	0.999
20Fe0.5Cu/MOR	14.739
20Fe0.5Cu0.5Ho/MOR	19.273
20Fe0.5Cu0.5Nd/MOR	16.469
20Fe0.5Cu0.5Ho/AC	0.115

a Determined from NH_3_-TPD.

b Determined from CO_2_-TPD.

**Figure 9:**
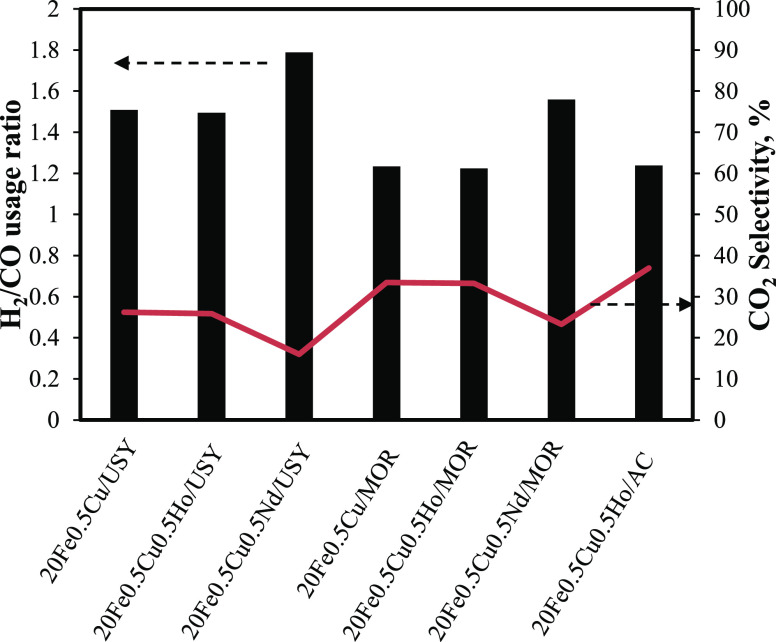
H_2_ to CO usage ratio and CO_2_ selectivity (%) of catalysts.

Usage ratios of H_2_ to CO of catalysts given in Figure 9 and CO_2_ selectivity (at the end of the 21 h of operation time) given in Table 5 are indicators of the extent of WGS reaction.^67^ The related CO_2_ selectivity vs TOS data of all catalysts is given in Figure S8. According to the reactions given below, FTS simply proceeds over reaction 1, whereas CO_2_ forms via water gas shift (WGS) reaction. Without WGS, hydrogen to carbon monoxide consumption rates would ideally be 2. However, this ratio will be lower than 2 upon the kinetics of WGS reaction.
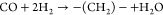
1
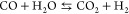
2
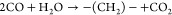
3

It is apparent that the usage ratios and CO_2_ selectivity of catalysts were in good agreement, demonstrating that the USY support is better in suppressing WGS activity of 20Fe0.5Cu catalysts. Among all catalysts, 20Fe0.5Cu0.5Nd/USY had the lowest CO_2_ selectivity, i.e., the lowest WGS activity. There is a general consensus that acidity is the key regarding the catalytic properties of zeolites in spite of the difficulty for quantitative assertion of structure–reactivity relations.^54^ As a plausible explanation, solid acidity might offer more favorable conditions for the WGS reaction. As shown in Figure S9, the increased acid site concentration leads to an increased CO_2_ selectivity.

Overall, it can be deduced that the olefin yield and selectivity can be increased for a catalyst with the optimum acid–base character. Benchmarking with AC-supported catalyst, the increased olefin yield and selectivity were ascribed to the role of zeolite-active metal-promoter combination. It can be claimed that these results indicate the bifunctionality of zeolite-supported catalysts in which paraffins formed on the active metal sites are dehydrogenated through acid catalyzed reactions on acidic sites of zeolites. On the other hand, AC-supported 20Fe0.5Cu0.5Ho seems to be an ideal catalyst for producing light paraffins together with methane for high-temperature FTS.

**Figure 10:**
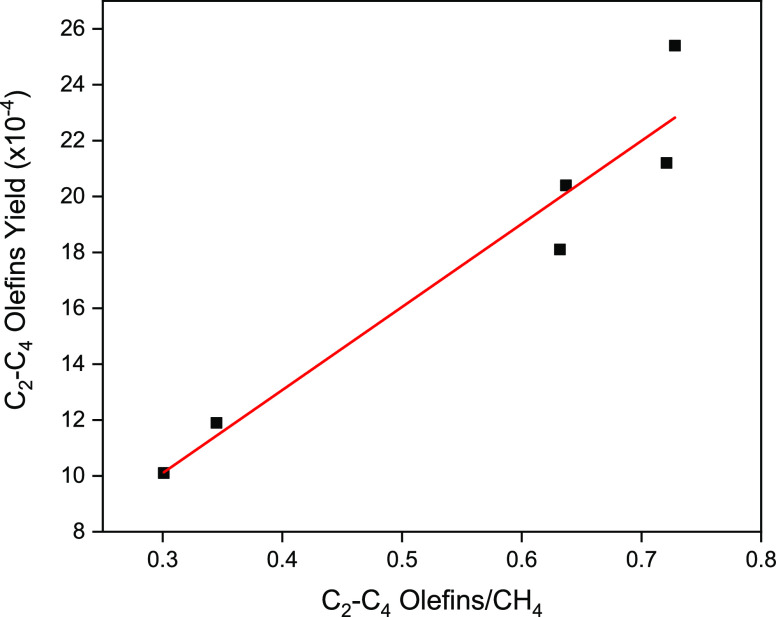
C_2_^=^-C_4_^=^/CH_4_ ratio vs light olefin yield data obtained using the P-CPA system.

Another important aspect of the P-CPA results is how well the C_2_^=^-C_4_^=^/CH_4_ ratio data correlates with that obtained in HT-CPA. The P-CPA data in Figure 10 show that there is a linear relationship between the C_2_^=^-C_4_^=^/CH_4_ ratio vs olefin yield. These data, obtained at close to realistic FT conditions, justify the use of C_2_^=^-C_4_^=^/CH_4_ ratio as a performance indicator. Calculated values of olefin/methane ratio on MOR- and USY-supported catalysts are given in Table S2 for both P-CPA and HT-CPA systems. As expected, when switching from HT-CPA to P-CPA analysis, the values changed. However, the relative performance of catalysts with respect to each other, i.e., the rank of catalyst performance, is the same in both systems, validating the use of HT-CPA in the rapid screening of FT-Olefin catalysts.

## 3 Conclusions

In this study, 24 catalysts were synthesized by co-impregnating Fe, Cu, and RE on USY and MOR supports. These catalysts were screened for their FT-Olefin performances using a high-throughput test system at atmospheric pressure. Regarding the C_2_^=^C_3_^=^/CH_4_ ratio as an indicator for light olefin production performance, six catalysts were selected to be tested at high pressure in a conventional test system. High-throughput analysis results correlated well with those obtained from the conventional high-pressure and high-temperature FT reaction system, validating the use of high-throughput techniques in screening of catalysts for FT-Olefin synthesis. Our experimental results, aligned with the literature, showed that the acidic/basic and structural properties of the zeolites dictate the FT-Olefin performance of the catalysts and that RE promotion enhanced C_2_^=^-C_4_^=^ selectivity. We conclude that the zeolite-supported catalysts prepared and tested in this study comprise more than one type of active site with both acidic and basic natures. Also, these catalysts acted as bifunctional catalysts where syngas was converted to paraffinic hydrocarbons on active metal sites, and these paraffinic hydrocarbons were dehydrogenated to olefins on acidic sites of the zeolites.

## 4 Experimental Methods

### 4.1 Catalyst Preparation

Supported Fe catalysts containing Cu and rare earth metals (RE) as first and second promoter, respectively, were prepared using the co-impregnation method. For support, ultrastable Y (USY) zeolite with a Si/Al ratio of 80 (Zeolyst, CBV780) and mordenite (MOR) with a Si/Al ratio of 20 (Zeolyst, CBV21A) were used. For FeCu/zeolite catalysts, Fe(NO_3_)_3_·9H_2_O (Merck, ACS grade) and Cu(NO_3_)_2_·3H_2_O (Merck, >99.5%) precursors were dissolved in deionized water, and the metal solution was then added to support. FeCuRE/zeolite catalysts were prepared using La(NO_3_)_3_·6H_2_O (Merck, for analysis), Ce(NO_3_)_3_·6H_2_O (Merck, extra pure), Nd(NO_3_)_3_·6H_2_O (Alfa Aesar, 99.9%), Ho(NO_3_)_3_·6H_2_O(Alfa Aesar, 99.9%), and Er(NO_3_)_3_·6H_2_O (Alfa Aesar, 99.9%) precursors. The mixed metal solution of Fe, Cu, and rare earth metals (RE) was then added to zeolites. Prepared mixtures were kept in an ultrasonic bath for 30 min; after this, the catalysts were dried at 100 °C overnight and calcined under dry air at 460 °C for 3 h. The prepared catalysts all containing 20 wt % Fe were denoted as 20Fe*x*Cu/USY, 20Fe*x*Cu/MOR (*x*: 0.5 and 1 wt %) and 20Fe*x*Cu*y*RE/USY, 20Fe*x*Cu*y*RE/MOR (*y*: 0.5 wt %). For comparison, an AC (Alfa Aesar)-supported 20Fe0.5Cu0.5Ho/AC catalyst was prepared by the same method. Finally, catalysts were crushed and sieved to the size range of 50–200 μm to be used in the experiments.

### 4.2 Catalyst Characterization

The BET surface area and the pore volume were determined by N_2_-adsorption at 77 K in a Micromeritics 3 Flex Surface Characterization Analyzer after outgassing the samples at 150 °C for 24 h under a vacuum condition. FT-IR experiments were performed on a Jasco-4000 FTIR spectrometer at room temperature by using the Praying Mantis accessory (Harrick). The phases of the fresh and spent catalysts were characterized by XRD (Shimadzu XRD-600). XRD patterns were obtained using Cu Kα radiation (λ = 0.15405 nm) and in 2–70° (2θ) range. Reducibility of catalysts was determined by H_2_-temperature programmed reduction (H_2_-TPR) experiments in a fixed-bed reactor. Prior to H_2_-TPR, 0.4 g of the catalyst sample was flushed in a N_2_ atmosphere at 350 °C and kept for 30 min to remove the adsorbed water on the surface. After cooling to 50 °C, 10 vol % H_2_/90 vol % N_2_ gas mixture was passed through the catalyst bed with a flow of 70 mL/min, and the temperature was increased to 800 °C at a rate of 10 °C/min. The TPR profiles were collected by both a micro-GC (Inficon, micro-GC Fusion) with a thermal conductivity detector (TCD) and a mass spectrometer (MS) (Stanford Research Systems, RGA 200). Temperature-programmed desorption of ammonia (NH_3_-TPD) was carried out in the fixed-bed reactor. The catalyst (0.2 g) was purged at 350 °C for 30 min under 60 mL/min He flow to remove the water from the surface. After the sample was cooled to 70 °C in He flow, NH_3_ adsorption was performed using a gas mixture of 5 vol % NH_3_/95 vol % He for 30 min. The sample was then purged again by He flow for 30 min to remove the physiosorbed NH_3_ from the surface. Subsequently, the sample was heated to 850 °C at the rate of 10 °C/min, and NH_3_ desorption peaks were recorded by MS. In determining the desorption peak of NH_3_, the MS peak at *m*/*z* = 16 instead of *m*/*z* = 17 was used because of the presence of a fragmentation peak of the desorbed water at *m*/*z* = 17.^68^ CO_2_-TPD experiments were conducted to determine the basicity of catalysts. The same procedure as in the NH_3_-TPD experiment was performed using a gas mixture of 5 vol % CO_2_/95 vol % He, and CO_2_ signals were recorded by the micro-GC. The amount of carbon formed over the catalysts during the FT synthesis was determined by a Vario MACRO cube analyzer using the ASTM D 4239-12 method.

### 4.3 High-Throughput Catalyst Performance Analyzer (HT-CPA)

The FT-Olefin performance of catalysts was measured using two different reaction systems: (i) the high-throughput catalyst performance analyzer (HT-CPA) at atmospheric pressure and (ii) the conventional pressurized catalyst performance analyzer (P-CPA).

**Figure 11:**
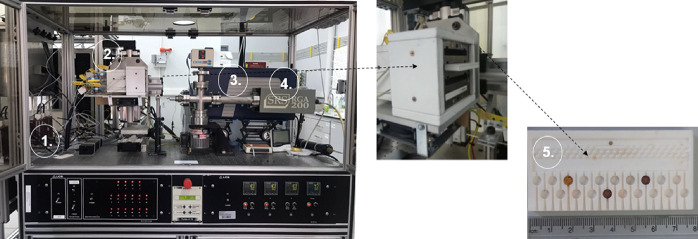
High-throughput catalyst screening system: (1) gas feeding system, (2) reactor block, (3) micro-gas chromatograph (micro-GC), (4) mass spectrometer (MS), and (5) reactor table.

Fast screening of performance of synthesized catalysts was carried out by a throughput catalyst performance analyzer (HT-CPA) at atmospheric pressure. Detailed information of the system has been described in detail elsewhere.^69,70^ To summarize, HT-CPA, shown in Figure 11, is a highly modular and versatile atmospheric pressure catalyst screening system that is completely computer integrated to enable the parallel testing of up to 80 distinct catalyst powders and/or pellets using array channel microreactor technology. It is composed of gas feeding, reactor, and analysis sections. The reactant gases are introduced through calibrated mass flow controllers. The reactor block comprises four reactor plates each of which includes 20 microreactors each with a diameter of 4 mm. The structure of the reactors allows for equal distribution of reactant gases to each and every microreactor. Owing to the micro design of reactors, the required amount of catalyst ranges from 5 to 20 mg with respect to the type/volume/density of the catalyst. Gas analysis at the microreactor outlet is accomplished by using a capillary sampling probe followed by an online quadrupole mass spectrometer (Stanford Research Systems, RGA 200) and a micro-gas chromatograph (Inficon, micro-GC Fusion). The micro-GC has two modules, RT-Q Bond and RT-Molecular Sieve 5A, and each is equipped with thermal conductivity detectors (TCDs). The run time of the micro-GC for FT products up to C_5_ is 3 min. The entire system is controlled by an automated software. This software enables control of temperature, inlet gas flow, gas sampling, and 3D movement of reactor block.

Prior to the reaction, catalysts were reduced with a gas mixture of 50 vol % H_2_ (Linde, 99.999%)/50 vol % N_2_ (Linde, 99.999%) at 350 °C for 4 h with a flow rate of 7 mL/min. Catalysts were subsequently cooled to a reaction temperature of 310 °C under inert N_2_ flow. Then, the reaction gas mixture consisting of 60 vol % H_2_/30 vol % CO (Linde, 95 vol % CO–5 vol % N_2_)/10 vol % N_2_ was fed to the system with a flow rate of 7 mL/min per microreactor. Effluent gas contents and compositions were analyzed using the micro-GC. Because of the configuration of the micro-GC, distinction between the signals from C_4_ olefins and paraffins cannot be achieved. Therefore, in HT-CPA, ethylene and propylene (C_2_^=^-C_3_^=^) are considered to represent light olefins. The mol C/g·h ratio of (C_2_^=^-C_3_^=^)/CH_4_ calculated from micro-GC analysis results was used to compare the performance of catalysts.

### 4.4 Pressurized Catalytic Performance Analyzer (P-CPA)

The catalysts that displayed high performance in screening were then analyzed at high pressure in a conventional pressurized catalyst performance analyzer (P-CPA), which is described elsewhere.^70^ The prepared catalyst (1 g) was mixed with quartz (Sigma-Aldrich, purum p.a.) of the same particle size as catalysts in a volumetric ratio of 1:1, and it was loaded in a fixed-bed reactor (i.d. = 10 mm; length = 800 mm). Prior to the reaction, catalysts were activated at 350 °C under a flow of 50 vol % H_2_/50 vol % N_2_ for 4 h with a flow rate of 50 mL/min. Then, the temperature was lowered to the reaction temperature of 310 °C under N_2_ flow, and reaction was performed with 50 mL/min (GHSV =1700 h^–1^) flow of 60 vol % H_2_/30 vol % CO/10 vol % N_2_ at 10 bar. Gas analysis was carried out using an online gas chromatograph (Agilent, GC 7820) equipped with a thermal conductivity detector (TCD) and flame ionization detector (FID) in which the columns were Carboxen and Alumina S, respectively.

The CO conversion (*X*_CO_) and product selectivity (*S*_Cn_) on carbon basis without CO_2_, hydrocarbon distribution on carbon basis, olefin yield (g C/g Fe·s) on carbon and weight basis, and FT yield were calculated using eqs 4–10 in which *F*, *n*_C_, and MW_C_ represent the mol/h, carbon number, and molecular weight of carbon, respectively.
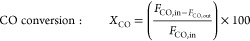
4
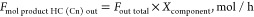
5

Hydrocarbon selectivity:
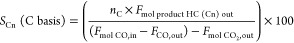
6
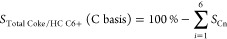
7

Hydrocarbon distribution:
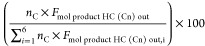
8

Olefin yield:
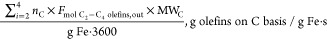
9

FT yield:
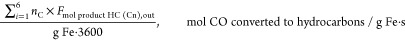
10

H_2_ to CO usage ratio was calculated on the basis of the ratio of consumed H_2_ (mol) to consumed CO (mol) as given in eq 11:
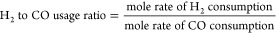
11

## Supporting Information Available

Additional characterization data of supports and catalysts; figures correlating selectivity vs TOS; selectivity vs acid sites; tables of acidic and basic site concentration on supports, HT-CPA vs P-CPA data comparison (PDF)
