# Supplementary material for: Effects of Rare Earth Metal Promotion over Zeolite-Supported Fe–Cu-Based Catalysts on the Light Olefin Production Performance in Fischer–Tropsch Synthesis
Source: ACS Omega. 2022 Dec 27;8(1):648–62. doi: 10.1021/acsomega.2c05795 (PMC9835664; doi:10.1021/acsomega.2c05795)
Supplement: Supplementary file 1 — ao2c05795_si_001.pdf [file ao2c05795_si_001.pdf]

Supporting Information

Effects of Rare Earth Metal promotion over Zeolite Supported Fe-Cu based Catalysts on the Light Olefin Production Performance in Fischer Tropsch Synthesis

*Utku Burgun[a,b], Hadi R. Zonouz[a,b], Hasancan Okutan[a,b], Husnu Atakul[a,b], Selim Senkan[b,c], Alper Sarioglan[a,b], Gamze Gumuslu Gur[a,b, *]*

[a] Istanbul Technical University, Chemical Engineering Department, 34467 Istanbul, Turkey

[b] ITU-SENTEK, ITU Synthetic Fuels and Chemicals Technology Center, 34467 Istanbul, Turkey

[c] University of California, Los Angeles, Chemical and Biomolecular Engineering Department, LA, USA

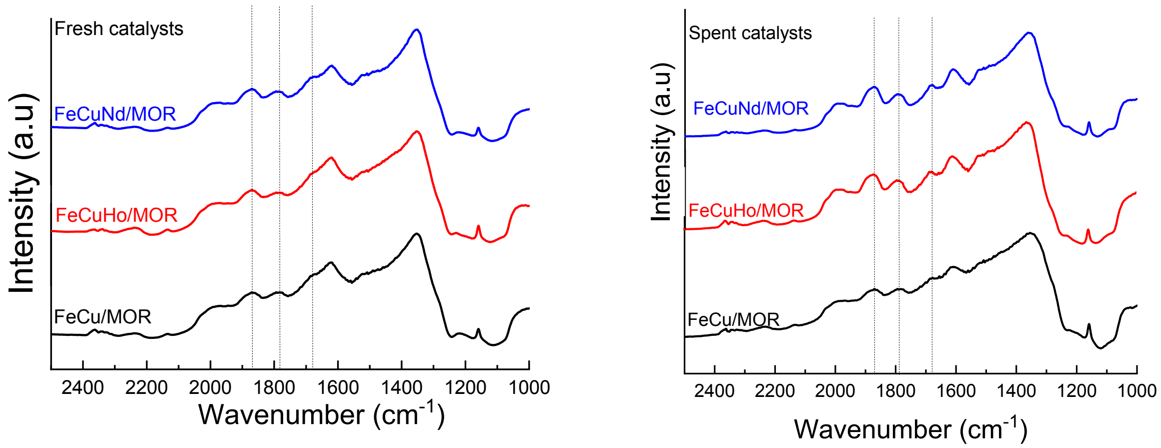

Figure S1. FT-IR spectra of fresh (left) and spent (right) MOR supported catalysts. Dotted lines indicate the bands where a significant change was observed. Weight percentages of the metal used are as follows: 20% Fe, 0.5% Cu, and 0.5% Ho or 0.5% Nd.

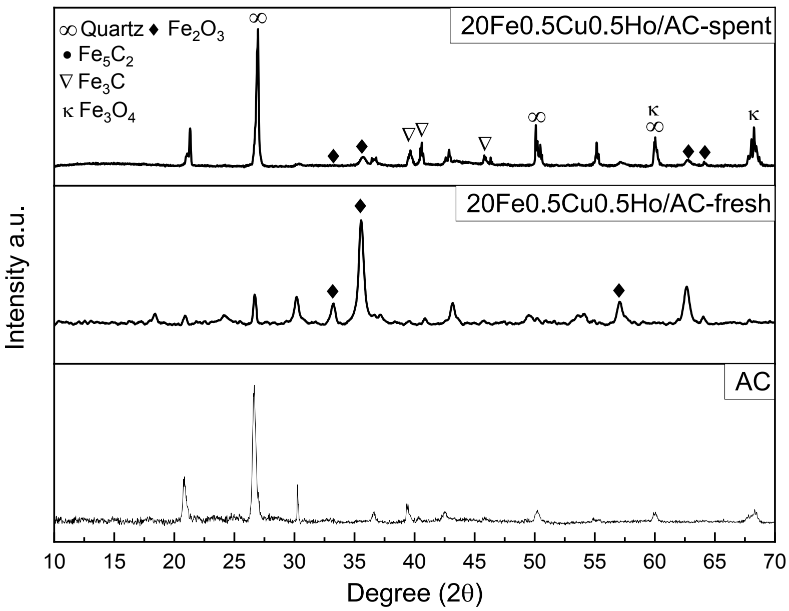

Figure S2. XRD profiles of the AC, fresh and spent 20Fe0.5Cu0.5Ho/AC catalyst.

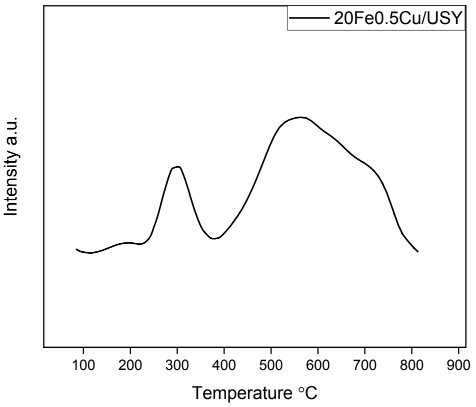

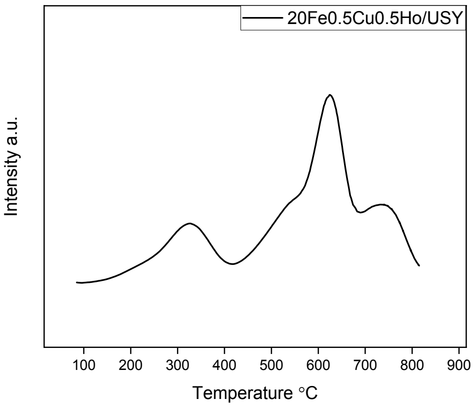

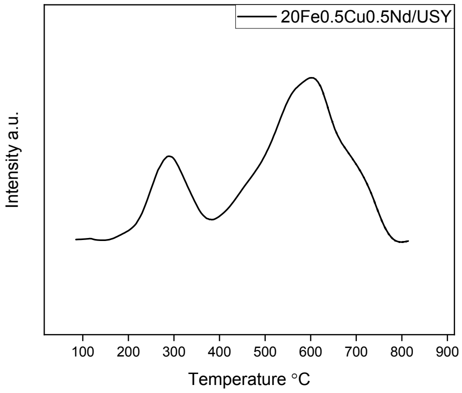
20Fe0.5Cu0.5Nd/USY
Intensity a.u.
100 200 300 400 500 600 700 800 900
Temperature °C

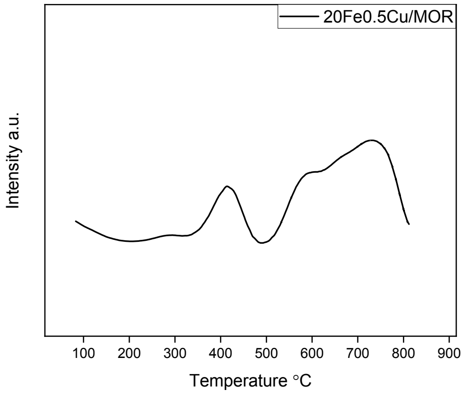
20Fe0.5Cu/MOR
Intensity a.u.
100 200 300 400 500 600 700 800 900
Temperature °C

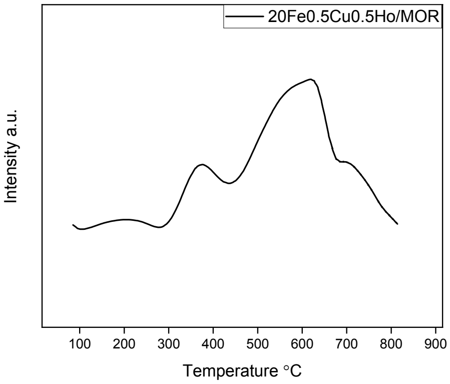
20Fe0.5Cu0.5Ho/MOR
Intensity a.u.
100 200 300 400 500 600 700 800 900
Temperature °C

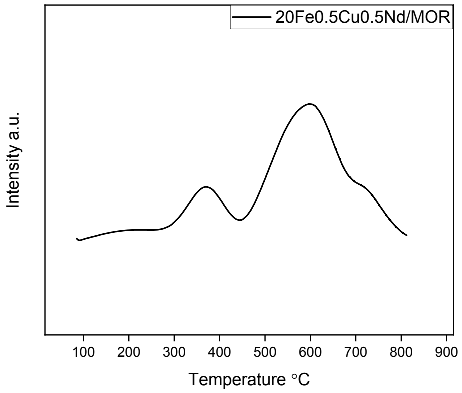
20Fe0.5Cu0.5Nd/MOR
Intensity a.u.
100 200 300 400 500 600 700 800 900
Temperature °C

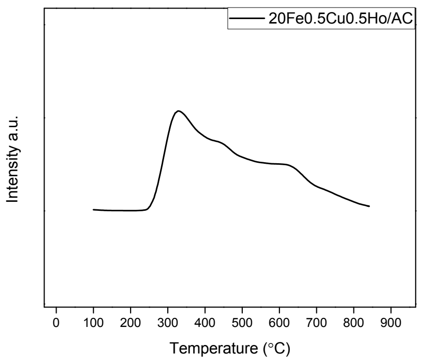

Figure S3. Individual H_2-TPR profiles of catalysts.

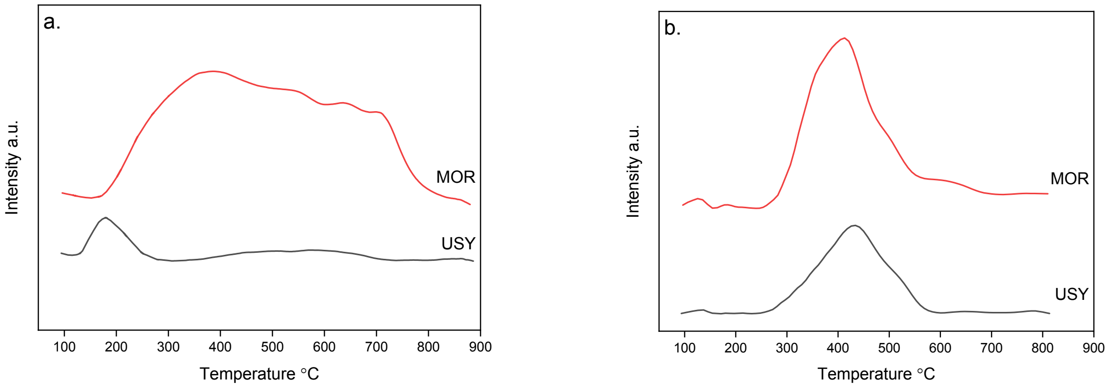

Figure S4. a. NH_3-TPD profile and b. CO_2 profile of USY and MOR supports.

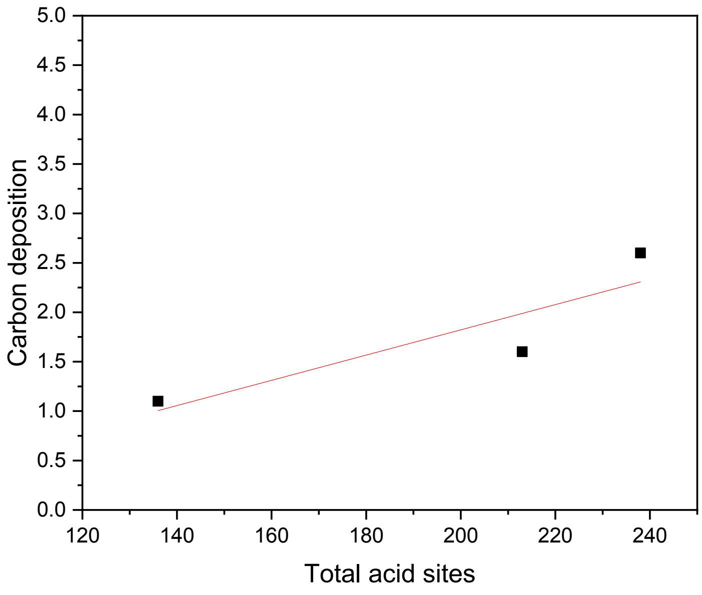

Figure S5. Carbon deposition versus total acidic sites of USY supported catalysts.

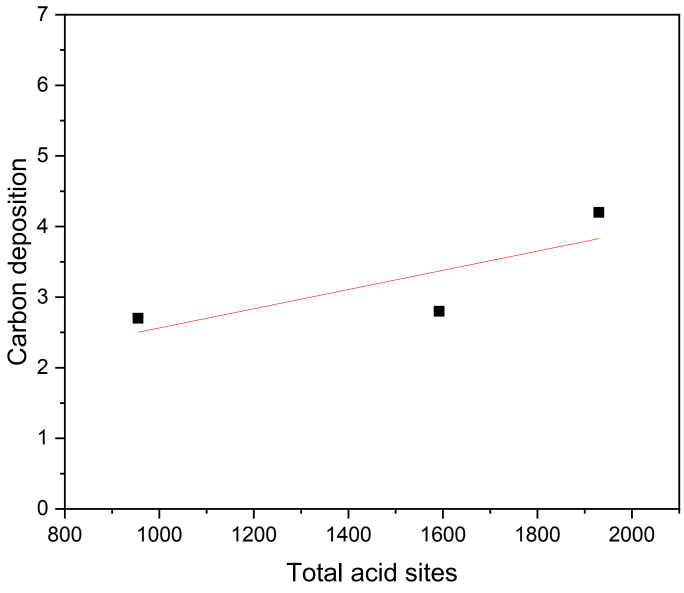

Figure S6. Carbon deposition versus total acidic sites of MOR supported catalysts.

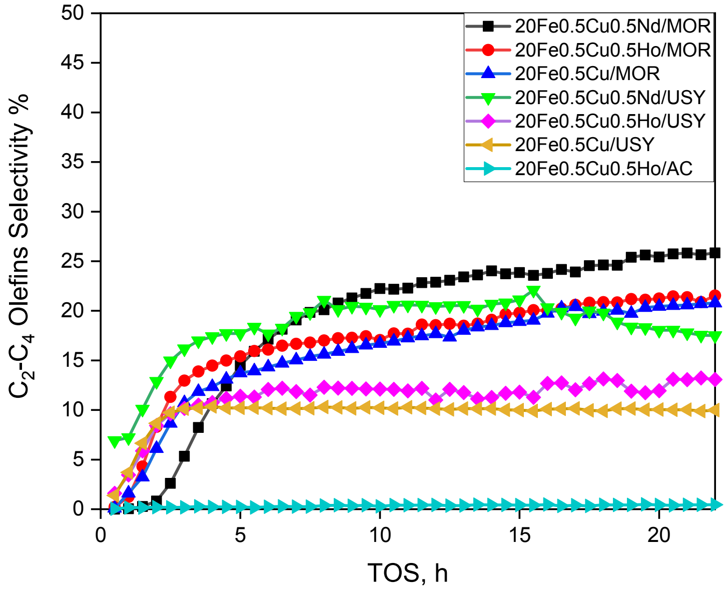

Figure S7. Light olefins selectivity of USY, MOR and AC supported catalysts versus time on stream (TOS)

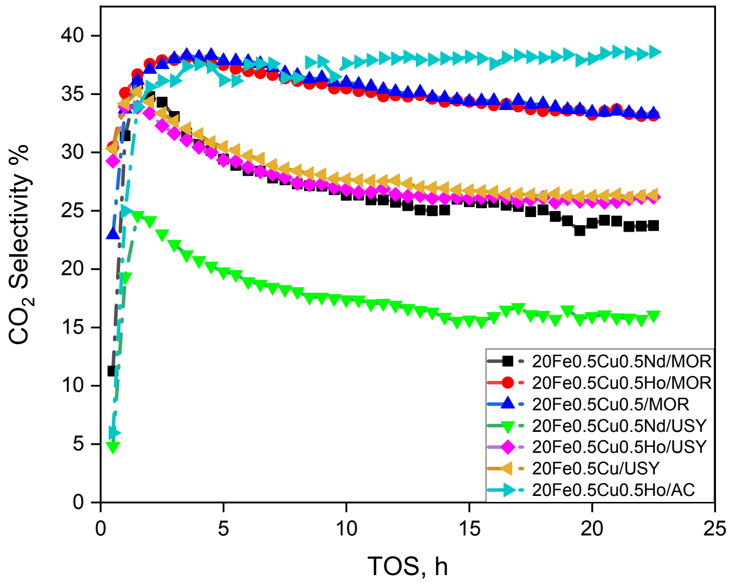

Figure S8. CO_2 selectivity of USY, MOR and AC supported catalysts versus time on stream (TOS)

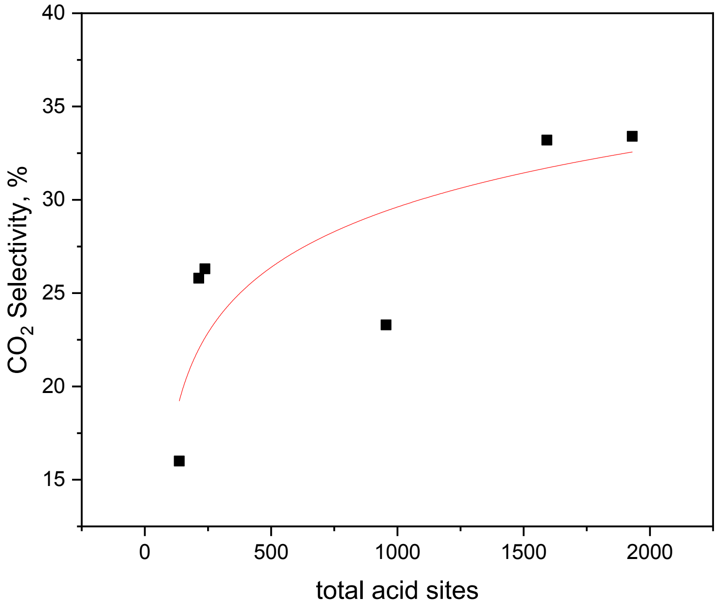

Figure S9. CO_2 selectivity of USY and MOR supported catalysts versus acidity

Table S1. The amount of total acidic[a] and basic site[b].

	Total Acidic Site (µmol)	Total Basic Site (µmol)
USY	502.0	3.6
MOR	3056	1.8
AC	n.p.[c]	253.9

[a] Determined from NH_3-TPD, [b] Determined from CO_2-TPD, [c] not performed

Table S2. FT-Olefin performance indicator Light Olefins/CH_4 ratio obtained on catalysts in HT-CPA and P-CPA. Results represent that the performance ranking of the catalysts correlate well in both systems despite the pressure difference.

Catalyst	HT-CPA	P-CPA
20Fe0.5Cu/USY	0.238	0.301
20Fe0.5Cu0.5Ho/USY	0.316	0.345
20Fe0.5Cu0.5Nd/USY	0.601	0.632
20Fe0.5Cu/MOR	0.351	0.637
20Fe0.5Cu0.5Ho/MOR	0.622	0.721
20Fe0.5Cu0.5Nd/MOR	0.963	0.728
